# Sperm Separation and Selection Techniques to Mitigate Sperm DNA Damage

**DOI:** 10.3390/life15020302

**Published:** 2025-02-14

**Authors:** Steven Fleming, David Morroll, Martine Nijs

**Affiliations:** 1 Discipline of Anatomy & Histology, School of Medical Sciences, University of Sydney, Sydney, NSW 2000, Australia; steven.fleming@coopersurgical.com; 2 CooperSurgical, 2750 Ballerup, Denmark; david.morroll@coopersurgical.com

**Keywords:** semen preparation, sperm DNA fragmentation, sperm selection, sperm separation

## Abstract

Semen preparation and sperm selection techniques exploit the morphological and physiological characteristics of sperm function, including motility, morphology, density, and maturity, as reflected by their cell-surface charge and the expression of hyaluronan receptors. The various methods employed have a common purpose of mimicing sperm selection within the female reproductive tract and, thereby, increasing the likelihood that oocytes will be fertilised by spermatozoa with intact nuclear DNA and a normal genome. Indeed, the paternal genome is relevant to embryonic genome activation and blastocyst development, and has a fundamental impact upon successful implantation, ongoing pregnancy and live birth. The clinical use of both well-established and some more recently developed techniques is discussed in this comparative clinical review of sperm separation from seminal plasma and selection for insemination.

## 1. Introduction

Approximately 15% of all couples suffer from subfertility, and 30–40% of infertility cases are believed to be due to a male factor. Male factor infertility has various aetiologies such as advanced age, lifestyle factors and obesity, exposure to infections and environmental toxins, testicular cancer, varicocele, congenital bilateral absence of the vas deferens, and various forms of testicular dysgenesis, resulting in impaired semen quality and sperm function. Though reproductive hormone assays can sometimes identify an endocrine disturbance, a semen analysis is regarded as the cornerstone of fertility diagnosis in the male. Repeated semen analysis can reveal various degrees of impairment in semen quality, categorised as moderate to severe oligozoospermia (O), asthenozoospermia (A), teratozoospermia (T), cryptozoospermia and azoospermia. These conditions are sometimes also linked to sperm DNA fragmentation and aneuploidy. Semen preparation and surgical sperm retrieval methods can mitigate most conditions of male factor infertility and enable treatment of an infertile couple using intrauterine insemination (IUI), or insemination by in vitro fertilisation (IVF) or intracytoplasmic sperm injection (ICSI), followed by embryo transfer. Despite difficulties with sterility in the past, advances in sperm preparation and insemination techniques have overcome most causes of infertility, contributing to the birth of over 12 million babies following medically assisted reproduction (MAR). Nevertheless, the efficiency and efficacy of sperm separation and selection partly determines the lab and clinical success of IUI, IVF and ICSI, and this is the focus of this narrative review.

Effective semen preparation methods should ensure efficient separation of spermatozoa from the seminal plasma and other inclusions that might otherwise indirectly inhibit fertilisation, such as moribund and immature sperm cells, leucocytes and bacteria. Conventional methods of sperm preparation include semen washing, swim-up and discontinuous density gradient centrifugation (DGC), the latter having been considered the ‘gold standard’ for decades. More recently, advanced sperm separation techniques have been developed to make the process less dependent upon operator time and expertise, less damaging, and more effective with respect to sperm DNA integrity and viability. Selection of DNA-intact spermatozoa during semen preparation is especially important since sperm DNA damage is associated with an increased rate of spontaneous abortion. Therefore, the quality of sperm separation methods will impact the clinical outcomes of IUI and IVF, and for ICSI it may be even more important, since the role of sperm selection by the oocyte and its investments are undertaken by the ICSI operator alone.

Sperm preparation techniques may be differentiated between those that separate spermatozoa from the seminal plasma and other constituents of the semen sample, and those that select individual spermatozoa from the initial sperm separation. Therefore, they can complement one another when used in combination. Sperm separation methods include swim-up, DGC, microfluidics, magnetic-activated cell sorting (MACS) and electrophoresis. Sperm selection methods include hyaluronan binding and motile sperm organelle morphology examination (MSOME), both of which are dependent upon micromanipulation. Sperm micromanipulation is essentially manual manipulation of spermatozoa under high magnification using microtools. There are various modifications of the ICSI technique, including physiological ICSI (using a PICSI^®^ dish or SpermSlow™), laser-ICSI, and piezo-ICSI, the latter being a particularly popular technique within Japan. Since sperm micromanipulation is highly operator-dependent and somewhat time-consuming, there is interest in developing more advanced techniques for sperm selection and micromanipulation, such as robotic ICSI and ICSI using photonic micropipettes. However, these futuristic techniques require further development prior to their introduction into clinical practice. Hence, this review will be limited to only those sperm separation and selection techniques which are currently routinely used within assisted reproduction laboratories.

## 2. Separation of Sperm by Swim-Up

### 2.1. Introduction

The sperm separation technique known as ‘swim-up’ is based upon the ability of spermatozoa with good motility to spontaneously migrate from a semen sample or a sperm pellet, placed at the bottom of a centrifuge tube or test tube, up into an overlaid sperm preparation or wash medium [1]. Due to limited sperm recovery following swim-up, this means of sperm preparation is best reserved for semen samples of higher quality, though swim-up is also recommended for the selection of motile spermatozoa in men with asthenozoospermia [2]. Generally, it is advisable that swim-up be performed on a semen sample (direct swim-up) rather than a sperm pellet, since washing and centrifugation of a semen sample can lead to the generation of reactive oxygen species (ROS) and peroxidative damage to the sperm plasmalemma [3]. Nevertheless, swim-up from sperm pellets has been suggested to yield a more concentrated sperm preparation in men with oligoasthenoteratozoospermia [4].

### 2.2. Technique

**Figure 1:**
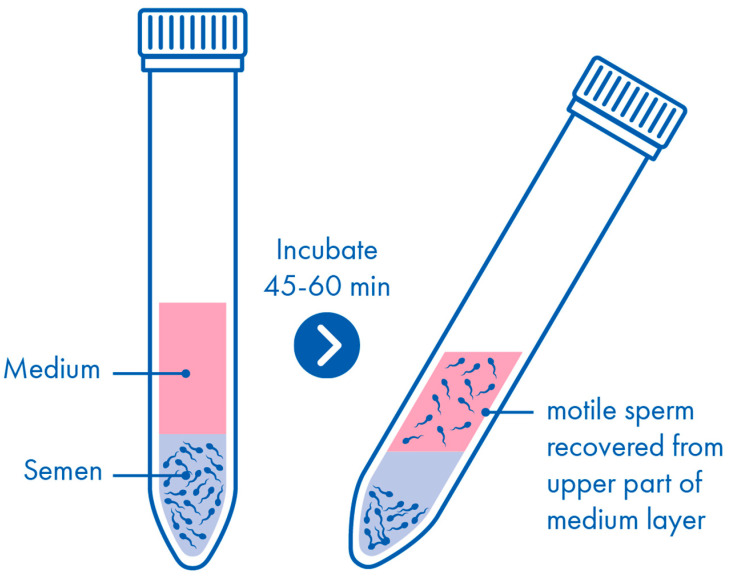
Swim-up.

Preparing a swim-up may seem deceptively simple but, in practice, requires extreme care to avoid disturbance of the semen sample since, otherwise, poorly or non-motile spermatozoa will be inadvertently mixed with the medium overlay. After ensuring that the semen sample is well mixed, 1 mL is placed at the bottom of a sterile conical centrifuge tube and is gently overlaid with 1 mL of sperm handling medium (Figure 1). Alternatively, 1 mL of sperm handling medium may be carefully underlaid with 1 mL of the semen sample. The tube is typically inclined at an angle of approximately 45° to increase the surface area at the interface between the semen sample and the sperm handling medium, and is placed into an incubator at 37 °C for 45–60 min. Taking care not to disturb the semen sample, the supernatant is then removed from the tube and is placed into a clean tube where it is washed with 1–2 mL fresh sperm handling medium and centrifuged at 300–500× *g* for 5 min. The supernatant may then be removed, leaving the final sperm preparation in approximately 0.5 mL of medium at the bottom of the tube.

### 2.3. Clinical Use

As might be expected, swim-up produces a higher percentage of progressively motile spermatozoa than DGC [5]. As determined using the two-tailed single gel electrophoresis assay and sperm chromatin dispersion test, although the swim-up method selects for spermatozoa devoid of double-stranded DNA damage, it is less effective than DGC in selecting against spermatozoa containing single-stranded DNA damage [6]. On the other hand, compared to DGC, spermatozoa prepared by swim-up have been observed to have fewer sperm head vacuoles [7]. Nevertheless, a recent meta-analysis could find no significant difference in clinical outcomes between the swim-up and DGC semen preparation methods [8].

### 2.4. Summary

The swim-up method provides a relatively simple and economical means of sperm preparation for IUI and IVF, though it is less effective for men with severe oligoasthenozoospermia due to its low recovery rate.

## 3. Separation of Sperm by Density Gradient Centrifugation

### 3.1. Introduction

The discontinuous DGC semen preparation technique was developed to mimic, in vitro, the natural selection of morphologically normal, viable spermatozoa by cervical mucus in vivo [9]. Naturally, the discontinuous gradient must remain stable in solution and cannot be toxic to spermatozoa, colloidal silica coated with silane or polyvinylpyrrolidone being a popular choice as a gradient material. Indeed, the colloidal silica particles help to cushion spermatozoa during centrifugation by virtue of their buoyant density, thereby minimising potential disruption of the sperm-nuclear chromatin. As its name implies, sperm separation using DGC essentially relies upon the greater density of morphologically normal spermatozoa, which possess a dense and homogeneous nucleus, those with morphological anomalies of the sperm head tending to be less dense and, therefore, not migrating as far down the gradient of colloidal silica of increasing concentration [10]. Therefore, DGC selects for spermatozoa of normal, density, size and shape, most of which also tend to be motile. Other constituents of semen such as seminal plasma, bacteria, debris, leucocytes, non-gametic cells, and immature sperm cells either fail to penetrate the colloidal silica or migrate to different densities of the discontinuous gradient. Hence, DGC tends to be the sperm separation method of choice for handling semen samples from patients with known infectious diseases. Removal of seminal plasma is necessary since some of its constituents, known as decapacitating factors, can stabilise the sperm plasmalemma, thereby preventing capacitation and hyperactivation from occurring. Otherwise, DGC is considered a more efficient sperm separation method than swim-up, with approximately 50% recovery rates of morphologically normal motile spermatozoa from normal semen samples having been reported [11]. Therefore, DGC is particularly suitable for sperm separation for IUI, IVF, or ICSI, even in men with oligozoospermia. In men with severe oligoasthenozoospermia mini gradients, using lower volumes than standard has proven a useful approach to DGC [12].

### 3.2. Technique

**Figure 2:**
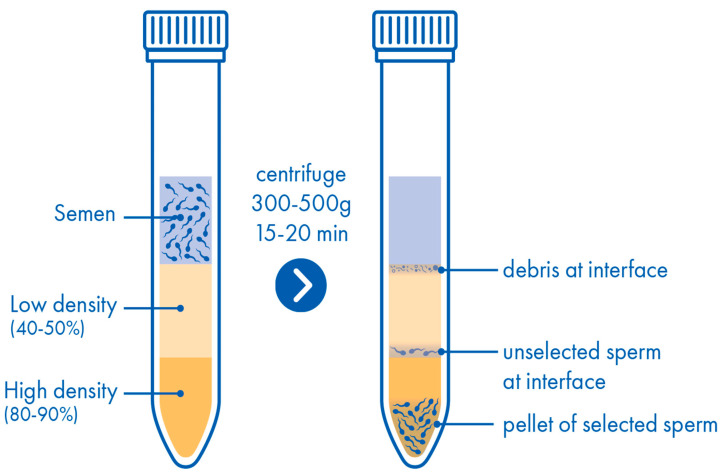
Density gradient centrifugation.

The desired concentrations of gradient material may either be purchased or prepared in advance of their use, making DGC adaptable for different qualities of semen samples or surgically recovered spermatozoa. Typically, 45% and 90% solutions of silane-coated colloidal silica are employed for standard semen preparation. If using 100% stock solutions of gradient material, a 90% isotonic gradient is simply prepared by diluting it with sperm preparation medium in a ratio of 9:1. An aliquot of the prepared 90% gradient may then be diluted 1:1 with sperm preparation medium to generate a 45% gradient (Alternatively, the 100% gradient material may be diluted with sperm preparation medium in a ratio of 9:11). To prepare a discontinuous gradient, 1 mL of the 90% gradient is placed into a sterile conical centrifuge tube and is gently overlaid with 1 mL of the 45% gradient, taking care to avoid mixing them (Figure 2). Then, the discontinuous gradient is gently overlaid with 1 mL of the premixed semen sample. The tube is centrifuged at 300× *g* for 20 min to separate the spermatozoa from the seminal plasma and other constituents of the semen sample. Taking care not to disturb the sperm pellet, the supernatant is removed, leaving approximately 0.5 mL of 90% gradient containing the sperm pellet at the bottom of the tube. The bottom 0.25 mL containing most of the spermatozoa is then transferred to a clean tube containing 5 mL of fresh sperm preparation medium where it is mixed and then centrifuged at 300× *g* for 5 min and this step is then repeated to ensure that the sperm pellet has been washed twice.

### 3.3. Clinical Use

Using prostatic zinc as a marker, DGC was found to reduce the contamination of the sperm preparation by soluble seminal components, compared with swim-up where a time-dependent diffusion of zinc into the medium overlay was observed [12]. On the other hand, compared with swim-up, DGC of normozoospermic semen samples has been observed to result in high levels of oxidative stress with increased capacitation, hyperactivation, and tyrosine phosphorylation [13].

### 3.4. Summary

Semen preparation by DGC yields a good recovery of morphologically normal motile spermatozoa. However, there are concerns that generation of ROS because of centrifugation may increase sperm DNA fragmentation and apoptosis via oxidative stress. Therefore, there remains a need for alternative methods of sperm separation that avoid the generation of ROS, thereby maintaining the integrity of sperm DNA.

## 4. Separation of Sperm Using “Microfluidic Chips”

### 4.1. Introduction

Microfluidics can play a significant role in studies of sperm motility as well as the selection of sperm for use in medically assisted reproduction. The accumulation of human sperm at, or near, surfaces or boundaries, together with the associated shift in pattern of motility and velocity, provide mechanisms by which sperm can be sorted and prepared for a full review; see [14].

In recent years, commercially produced “microfluidic chips” have been introduced that offer a very effective and efficient way of preparing sperm. Though often referred to in the scientific literature as microfluidic devices, these sperm separation devices (SSDs) do not use rheotactic, thermotactic, or chemotactic devices to sort sperm, but rely on motility-based selection. This is effected using a membrane with pores that separates the semen from clean medium; the most motile sperm can swim through the pores and may be harvested for use in treatments.

**Figure 3:**
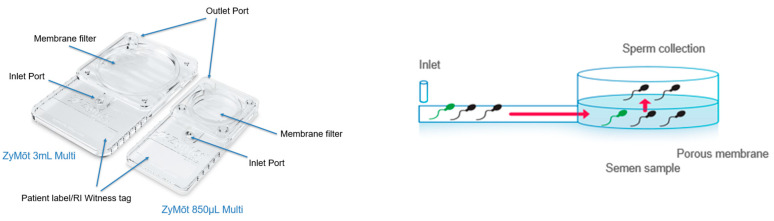
The ZyMõt™ Multi 850 µL and 3 mL devices.

This technology has been made available in the form of the two different capacity ZyMōt devices (Figure 3; CooperSurgical, Trumbull, CT, USA) which were also produced until recently under the name FERTILE**^®^** Chip. A third version, the ZyMōt ICSI SSD, does not use a membrane with pores, and instead uses microchannels. This has proved to be an effective approach (Quinn et al., 2018) but will not be discussed. Similar devices, such as the LensHooke^®^ CA0 (Bonraybio, Taichung City, Taiwan) and SwimCount™ Harvester (MotilityCount, Valby, Denmark) have also come to market and, while studies report similar findings on motility and SDF [15,16], clinical data are, as of yet, missing. The focus of the remainder of this section will therefore be on the ZyMōt devices.

### 4.2. Technique

**Figure 4:**
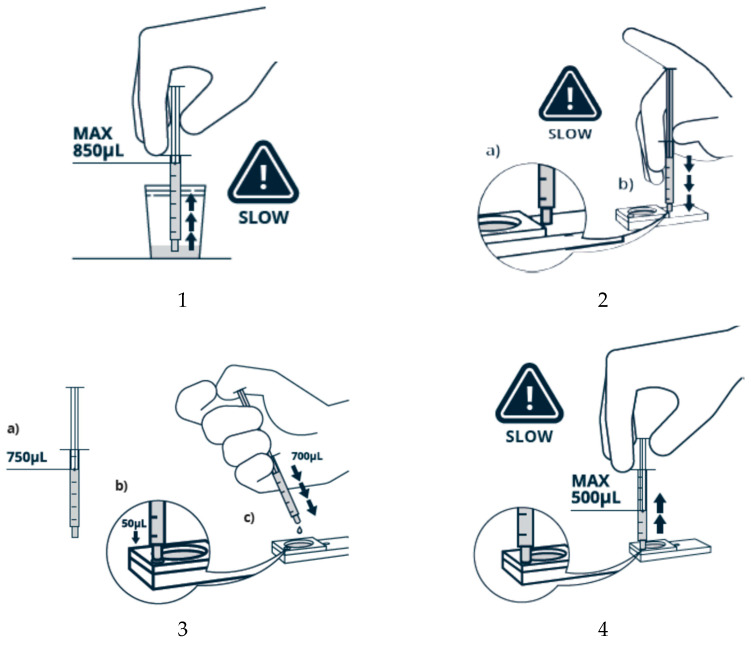
How to use the ZyMõt™ device: 1. Draw semen sample; 2. Inject sample (**a**) Achieve seal. (**b**) Slowly inject sample; 3. (**a**) Add 750µL of media. (**b**) Prime outlet channel. (**c**) Cover membrane surface; 4. After incubation, slowly aspirate a maximum of 500 µL.

Both ZyMōt™SSDs (Multi 850 μL and Multi 3 mL) are prepared in the same way, as described in Figure 4, but using different volumes.

In essence, a fixed volume of sample is injected, via the inlet port, into the chamber below the membrane, avoiding bubbles. A fixed volume of appropriate medium is then carefully injected into the outlet port before fully covering the upper surface of the membrane. The device is then incubated for 30 min at 37 °C, with steps being taken to ensure that evaporation is limited. To harvest the prepared sperm, a clean syringe is attached to the outlet port and the recommended volume aspirated (500 μL for the Multi 850 μL; 1.0–1.5 mL for the Multi 3 mL) and transferred to a suitable clean culture tube. For conventional IVF (cIVF), an additional wash step is needed. The medium used can be HEPES-/MOPS-buffered, in which case incubation should be in an un-gassed incubator, or a bicarbonate-buffered medium when the device is incubated in a CO_2_-enriched atmosphere. Laboratories can choose which fits best into their usual practises.

The additional wash step for cIVF was introduced after reports of lower/failed fertilisation and is thought to remove components of seminal plasma, possibly decapacitation factors, that migrate through the pores of the membrane with the motile sperm (ZyMōt Fertility, internal data). It is not necessary when performing ICSI or IUI with the prepared sample.

In addition to the significantly greater progressive motility and lower SDF than other preparation methods, the use of ZyMōt SSDs has fewer handling steps, is technically easier, provides more consistent results, and permits samples to be processed contemporaneously.

It is important to use the ZyMōt SSDs in suitable cases only: given the requirement for motile sperm, some cases may have too low a motile sperm count, with a nominal threshold of a total motile count of 1 million sperm. In addition, data are not yet available for use in serodiscordant couples (where the male partner may potentially transmit a viral infection), bacteriospermia, or non-ejaculated samples.

### 4.3. Clinical Use

The concentration of progressively motile sperm remains the single most predictive parameter for sperm quality regarding clinical outcomes [17]. Motility, per se, is linked to genetic integrity with several genetic causes of reduced motility [18], and an association between sperm motility and SDF [19,20,21]. There appears to be a consensus that ZyMōt preparation results in higher motility and lower SDF [22,23,24,25] compared with other methods, and it is a logical extrapolation to assume that this might, in turn, support better clinical outcomes.

Most of the data on fertilisation pertains to the use of ICSI so perhaps an impact of preparation method would not be anticipated.

Fewer data are available for cIVF. A recent study [26], however, supports the use of the ZyMōt SSD provided the additional wash step is performed.

**Figure 5:**
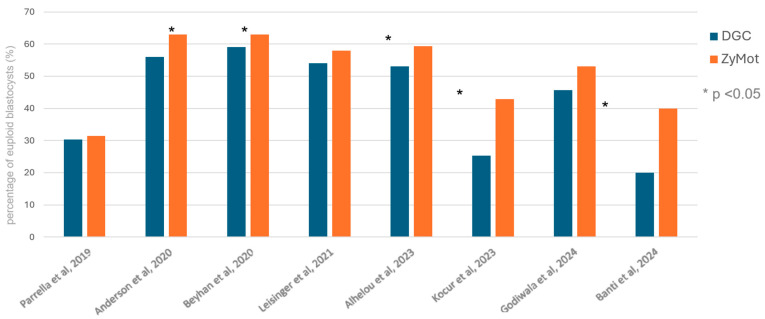
The rate of euploidy in blastocysts according to sperm preparation method [22,28,29,30,31,32,33,34].

Evidence for improved blastocyst formation rate after sperm preparation using the ZyMōt SSD (or the FERTILE^®^ Chip) is conflicting [22,27,28,29]. Similarly, in PGT-A cycles, the use of the ZyMōt SSD may increase rates of euploid blastocysts [27,28,30,31,32,33] but this is not a universal finding [22,29,34] (Figure 5). Given a raft of studies [35,36] linking SDF with incidence of aneuploidies in human embryos, the lower SDF post-preparation with the ZyMōt SSD might be beneficial.

The impact on rates of implantation and pregnancy after IVF/ICSI is somewhat unclear with reports of better outcomes [37] not being universal [38], though no studies have reported a negative impact on clinical outcomes. There may be a positive impact on pregnancy rates after IUI [39,40] though not all studies show this [41]. Data are, as yet, lacking for a possible impact on miscarriage rates, but there is a clear link between progressive motility, SDF, and recurrent miscarriage [42,43], both of which ZyMōt SSD has been shown to improve.

Overall, more data are needed to determine an impact on pregnancy rates across different treatment modalities, but the possible improvement in euploidy hints at more useable blastocysts that could potentially give better cumulative chances of conception, especially in selected cases such as men with high levels of SDF.

### 4.4. Summary

“Microfluidic” devices, such as the ZyMōt SSDs, select sperm based on motility which is, itself, a reliable marker of sperm health. Higher progressive motility and lower SDF offer the promise of better sperm for use in IUI, IVF, and ICSI leading to better outcomes. Though reports of better clinical outcomes are available, more data are needed to confirm these whilst also increasing our understanding of which patients might benefit most. The ZyMōt SSDs do however mean fewer handling steps, making sperm preparation technically easier, more reproducible, and reducing risks.

## 5. Separation of Sperm Using Magnetic-Activated Cell Sorting

### 5.1. Introduction

The acronym, MACS, defines a novel means of separating apoptotic spermatozoa from a semen sample using a column internally coated with annexin V conjugated to paramagnetic microbeads, held in place by magnets. Annexin V binds to various phospholipids, acting as an early event during sperm apoptosis, with the externalisation of phosphatidylserine across the sperm plasmalemma. Since apoptosis is closely associated with sperm DNA fragmentation, the use of MACS should theoretically increase the percentage of DNA-intact spermatozoa within the final sperm prep used for IUI, IVF or ICSI. Sperm separation using MACS is suitable for both freshly produced and cryopreserved semen samples.

### 5.2. Technique

Typically, MACS is combined with DGC since it is unable to separate spermatozoa from seminal plasma by itself. Effectively, annexin V acts as a biomarker for apoptotic spermatozoa, and as such may be used to separate them from the semen sample by retaining them along the walls of the column, non-apoptotic spermatozoa within the semen sample being allowed to pass through the column.

### 5.3. Clinical Use

Following MACS, before or after the cryopreservation of semen samples, non-apoptotic spermatozoa have been found to have improved rates of cryosurvival and motility [44]. However, in an RCT of sperm separation with swim-up versus swim-up followed by MACS, in oocyte donation cycles with unselected males, no significant difference was observed in clinical outcomes, including miscarriage rates [45]. Even in men with high rates of sperm DNA fragmentation, where MACS might be expected to be beneficial, there have been conflicting reports. A retrospective cohort study, comparing DGC versus DGC plus MACS, failed to demonstrate any statistical difference in live birth rates in couples undergoing ICSI using autologous or donor oocytes, irrespective of whether the men had moderate (≥30%–<50%) or high (≥50%) levels of sperm DNA fragmentation though, remarkably, there was no incidence of miscarriage following MACS [46]. In another retrospective study of patients with high (>20%) levels of sperm DNA fragmentation, DGC followed by MACS was found to significantly reduce miscarriage rates in autologous ICSI cycles, increase pregnancy rates in oocyte donation cycles, and significantly increase live birth rates in both autologous and donor cycles [47]. In a more recent study in patients with teratozoospermia, DGC followed by MACS, versus swim-up or DGC, alone or in combination, was shown to yield a significantly higher percentage of morphologically normal spermatozoa with condensed chromatin and intact sperm DNA, resulting in significantly higher cleavage, implantation, and pregnancy rates [48].

### 5.4. Summary

The evidence supporting the use of MACS is generally encouraging, though it would seem to be of greater benefit to those couples with male factor infertility. Consistent with the known relationship between sperm DNA fragmentation and miscarriage, the reported decrease in miscarriage rates following sperm preparation using MACS makes biological sense. However, larger RCTs are required to prove the benefit of this method of sperm preparation.

## 6. Electrophoretic Sperm Separation

### 6.1. Introduction

Electrophoresis exploits the differential net negative electric charge imparted by the glycocalyx of the sperm plasmalemma. The glycocalyx of normal mature spermatozoa acquires sialic acid residues during epididymal transit, including CD52, which is a highly sialilated glycosylphosphatidylinositol-anchored protein located on the sperm plasmalemma. The magnitude of the cell–cell transfer of CD52 may be dependent upon the negative charge associated with the sperm plasmalemma and may reflect normal spermatogenesis since its expression is significantly correlated with normal sperm morphology and capacitation.

### 6.2. Technique

Electrophoresis separates spermatozoa from semen by virtue of differences in surface charge due to the differential presence of sialated proteins on the sperm plasmalemma. Platinum-coated titanium mesh electrodes apply an electric potential to attract negatively charged spermatozoa across a 5 µm polycarbonate separation membrane towards the anode [49]. Hence, spermatozoa are selected based on both their normal morphology and their electric charge within just 6 min, only morphologically normal spermatozoa with low levels of sperm DNA damage passing through the separation membrane [50].

### 6.3. Clinical Use

Proof of principle for electrophoretic sperm separation was first demonstrated in an ICSI case report [51]. Subsequently, a prospective, split-sample, split-cohort, controlled trial of sperm separation by electrophoresis versus DGC showed that comparable rates of fertilisation, cleavage and embryo quality could be achieved in both IVF and ICSI patients [52]. The percentage of negatively charged sperm cells following electrophoresis has been found to be positively associated with the fertilisation rate, blastocyst development, implantation and clinical pregnancy rates [53]. Although the recovery rate following electrophoresis is lower than that with DGC, several studies have demonstrated that electrophoresis yields spermatozoa with significantly less oxidative DNA damage [54,55].

### 6.4. Summary

Electrophoretic sperm separation is a rapid technique that is able to yield a population of spermatozoa with low levels of DNA damage in less than 10 min. It compares favourably to DGC in terms of both efficiency and efficacy.

## 7. Selection of Sperm Using Hyaluronan Binding: Physiological ICSI (PICSI) and SpermSlow

### 7.1. Introduction

In the usual practice of ICSI, sperm are visually selected for injection based on their motility (primarily as a marker of cell viability) and morphology. However, this approach does not reflect the genomic integrity of the sperm and the paternal contribution to the zygote. It is known, for example, that morphologically normal sperm from men with oligoasthenoteratozoospermia have an elevated risk of aneuploidy [56]. To address this, additional techniques for de-selecting poor sperm and maximising the chance of choosing a genetically healthy sperm have been explored, including IMSI [57] and hyaluronan binding [58].

Hyaluronan is a major component of the *cumulus oophorus* as well as cervical mucus and follicular fluid [59] and mature sperm carry hyaluronan receptors [60], whilst immature sperm do not exhibit binding properties. Mature sperm exhibit high DNA chain integrity [61], a normal frequency of chromosomal aneuploidies and provide a paternal contribution to the zygote comparable to that of sperm selected by the zona pellucida during natural fertilisation [62]. Importantly, a reduced incidence of DNA fragmentation is seen in sperm that bind hyaluronan compared with those in neat semen or prepared sperm [58,63]. This is of particular significance given the possible association between sperm DNA fragmentation and impaired reproductive success, especially due to early pregnancy loss [17,64,65].

The unreliability of selection based on motility and morphology, plus the putative advantage of deselecting DNA-damaged sperm, suggest there should be clinical benefits in using hyaluronan binding as an additional screening procedure. This can be achieved by using one of two methods: the PICSI^®^ dish, in which hyaluronan dots are dried on the inner surface of a culture dish, and SpermSlow™ which has a high concentration of hyaluronan in solution.

### 7.2. Techniques

#### 7.2.1. PICSI^®^ Sperm Selection Device

**Figure 6:**
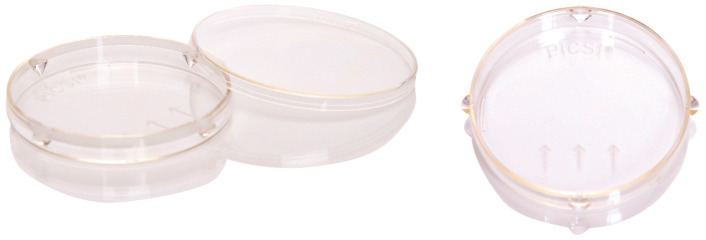
The PICSI dish showing the arrow heads indicating the location of hyaluronan dots.

The PICSI^®^ Sperm Selection Device (CooperSurgical Fertility Solutions, Måløv, Denmark) is a polystyrene culture dish with three dots of hyaluronan dried on the inner surface, indicated by arrow heads embossed on the bottom exterior surface of the dish (Figure 6).

Use of this device requires the hyaluronan dots to be rehydrated using an appropriate holding medium (typically HEPES-/MOPS-buffered) containing at least 5 mg/mL human serum albumin. The dish is then flooded with oil and the dish incubated while the hyaluronan dots swell. Prepared sperm can then be added at the edge of the rehydrating drop. Sperm binding can be seen within 5 min, in many cases, though some dots can take up to 30 min (see Instructions for Use at https://fertility.coopersurgical.com/dishes/picsi-dish-for-sperm-selection/) (accessed on 9 February 2025). It is recommended that sperm are added in a volume of medium equal to, or greater than, that used to hydrate the drop in order to ensure rapid mixing and delivery to the hyaluronan dot. However, it is the opinion of the authors that it is advantageous in most cases to add the sperm at the tip of the elongated droplets covering the hyaluronan dot such that motile sperm must migrate through the medium droplet to reach the hyaluronan dot. This mimics the approach often used in PVP and acts as a secondary selection mechanism, and controls the rate at which sperm bind; this is an important consideration, since overpopulation of the dot can make the selection and handling of individual sperm more difficult. Additionally, the volume of sperm suspension added to each drop can be varied, thus delivering differing numbers of sperm; this allows sperm to be recovered first from the drop with the most sperm added, and then from the second most, and finally from the drop with the least sperm. In this way, sperm can be collected over an extended time period before the microdot becomes congested. This is demonstrated in the manufacturer’s video at https://www.youtube.com/watch?v=bzF4rv-bDWQ or https://fertility.coopersurgical.com/dishes/picsi-dish-for-sperm-selection/ (accessed on 9 February 2025).

The official protocol involves preparation and use of the dish at temperatures below 30 °C as sperm velocity may overcome the binding force at higher temperatures. In a clinical setting, however, labs may prefer to use it at 37 °C so they can inject eggs in the same dish; indeed, the manufacturer states that the PICSI^®^ dish will typically reach 33 °C when on a heated stage set at 37 °C, but “at 33 °C or even at 37 °C, many bound sperm will remain available for selection”.

In use, the collection of the hyaluronan-bound sperm is easily performed by positioning the tip of the ICSI micropipette next to the sperm and gently sucking fluid into the pipette, drawing in the sperm. Sperm for injection must also be assessed based on morphology before breaking of the tail membrane as for routine ICSI.

#### 7.2.2. SpermSlow™

SpermSlow™ is a semi-viscous medium with a high concentration of hyaluronan. Though sometimes used simply as an alternative to PVP, SpermSlow™ allows the selection of mature; healthy sperm by their ability to bind with hyaluronan in the same way as the PICSI^®^ dish. However, the hyaluronan is in solution (forming a three-dimensional net) rather than coating the dish surface, so those sperm-expressing receptors bind with the hyaluronan and lose their progressive motility. This means selection is a little counterintuitive, as it is the sperm that swim freely that are ignored and those that become less motile that are chosen

**Figure 7:**
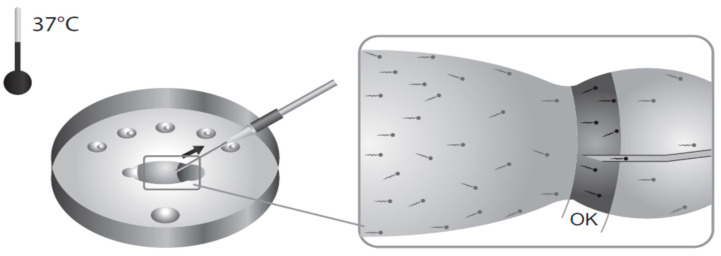
The set up used for SpermSlow indicating the interface with the bridging drop where sperm are selected.

After dispensing oocyte holding drops, sperm selection is facilitated by dispensing a drop of SpermSlow™ and a drop of the final prepared sperm suspension connected with a bridging drop of buffered handling medium in the laboratory’s standard ICSI dish before flooding with oil. The bridging drop allows sperm selection based on motility but also allows easy observation of binding at the interface between the bridging drop and SpermSlow™ (Figure 7). In cases with extremely poor sperm motility or numbers, the bridging drop can be omitted, and the edge of the sperm suspension drop simply blended with the SpermSlow™ drop. This is demonstrated in the manufacturer’s video at https://www.youtube.com/watch?v=TBD1yIwvWkw&t=119s or https://fertility.coopersurgical.com/art_media/spermslow/ (accessed on 9 February 2025).

### 7.3. Clinical Use

The selection, based on hyaluronan binding, of more mature, genetically competent sperm for injection into the oocyte should yield improved outcomes. It would also be reasonable to assume that the two techniques select sperm in the same way, and as such should have the same outcomes. Indeed, Parmegiani and colleagues (2012) [66] performed a prospective, randomised trial that demonstrated that fact.

Embryological endpoints show variable results when using PICSI or SpermSlow™. Several small studies have suggested higher rates of fertilisation when using hyaluronan binding selection (HABS) [67,68,69] though this is not a universal finding [63,70]. A small time-lapse study of sibling oocytes randomised to sperm selection with SpermSlow or in PVP suggested lower abnormal fertilisation and higher normal fertilisation [71], but embryo development at day 3 was not affected. In contrast, Parmegiani et al. (2010) [58] found a greater proportion of top-grade embryos. Alegre et al. (2016) [72] found significant improvements in embryo quality using PICSI, yet the same group [73] later reported no differences in embryo quality between PICSI and standard ICSI, though still suggesting that PICSI is advantageous in terms of cumulative pregnancy rates.

Similarly, studies are inconsistent in terms of demonstrating improved implantation or pregnancy rates with some suggesting better outcomes [58,74,75,76,77,78,79] though not always reaching statistical significance. Majumdar and Majumdar (2013) [70] looked specifically at patients with unexplained infertility, with a trend towards better clinical pregnancy rates and lower pregnancy loss, though numbers of patients were relatively low. Contrasting with an earlier study [68] that found no improvement in outcomes using PICSI for male factor infertility patients, another group [78] reported better clinical pregnancy rates and suggested that teratozoospermic patients benefited most.

Perhaps the most interesting association is between hyaluronan binding and early pregnancy loss. Given the data linking sperm DNA fragmentation and miscarriage [17], one might suspect that the apparent deselection of DNA-damaged sperm using hyaluronan binding [58] could reduce the risk of early pregnancy loss. A multi-centre study by Worrilow and collaborators [75] involving 802 patients observed higher implantation rates in the PICSI group, but these were not statistically significant; the study was halted prematurely and so it is impossible to know whether a fully powered study would have confirmed this. However, the pregnancy loss rate was markedly reduced when PICSI was used in partners of men with low proportions (<65%) of sperm that bound to hyaluronan, whether in the initial, unprocessed semen (3.3% vs. 15.1%; *p* < 0.05) or in the final sperm preparation (0% vs. 18.5%; *p* < 0.05). The authors concluded that determining the % sperm that bind hyaluronan, and then offering PICSI to those with 65% or fewer, might provide a balanced approach to using hyaluronan binding judiciously. In a much larger study in the UK [80] (HABSelect trial, 2772 couples, presenting with a large range of aetiologies, were assigned randomly to PICSI or standard ICSI; the primary endpoint used was term livebirth rate, which, though slightly higher, was not significantly improved (27.4% vs. 25.2%; *p* = 0.18). Conversely, the miscarriage rate was significantly reduced (4.3% vs. 7.0%; *p* = 0.003) but, unlike the Worrilow study [75], a subgroup analysis [81] indicated that this was not associated with the hyaluronan binding score, but was associated more with female age.

### 7.4. Summary

The ability of sperm to bind with hyaluronan is associated with maturity and greater genetic integrity. This is reflected in the reduced levels of DNA fragmentation when methods of sperm selection using hyaluronan binding are employed [58]. The fact that this does not consistently result in improved clinical outcomes is evident, though some reports are certainly encouraging. Reports of better outcomes in selected groups (for example, male factor cases [78] may indicate that patient selection is key. Logically, this might be based on not just aetiology but a screening test to identify men with lower levels of sperm-hyaluronan binding as demonstrated by Worrilow et al. (2013) [75], though this was not reproduced in the HABSelect study [81]. There does, however, appear to be a positive impact on reductions in miscarriage rates, possibly associated with the deselection of DNA-damaged sperm. The mechanistic analysis undertaken as part of the large HABSelect study concluded that “PICSI was protective against miscarriage and that female age was the strongest indication for PICSI efficacy in this regard”.

## 8. Selection of Sperm by Motile Sperm Organelle Morphology Examination

### 8.1. Introduction

The physiological rationale of MSOME is that sperm ultra-morphology may be correlated with spermatogenesis and sperm maturity. Various sperm structures may be viewed under very high magnification to reveal any abnormalities, particularly within the acrosome and nucleus [82,83]. However, due to a lack of adequately powered RCTs, there is no consensus regarding clear indications for intracytoplasmic morphologically selected sperm injection (IMSI). Furthermore, the theoretical basis of MSOME is complicated by the normal physiological appearance of vacuoles during spermatogenesis, capacitation and the acrosome reaction [84,85]. Such confusion and lack of hard scientific evidence for the benefit of IMSI has brought some to question its validity in clinical practice [86]. Subsequently, classification of sperm head vacuoles has been revised according to their correlation with genomic integrity, with their location and depth within the nucleus becoming more relevant than their size or number [87].

### 8.2. Technique

Application of MSOME requires a system comprising an inverted microscope fitted with differential interference contrast (DIC), 60× or 100× oil objective lenses, a micromanipulation rig, and an appropriate digital video imaging system with image analysis software. To achieve good resolution at that magnification, the NA of the objective lens needs to be high, and since bubbles within plastic ICSI dishes can interfere with optical resolution, it is recommended to use glass bottom Petri dishes (e.g., Willco wells BV, Amsterdam, The Netherlands). Sperm aspiration into an injection pipette may facilitate MSOME since it provides greater control of sperm motility and allows both sides of the sperm head to be viewed. Performing MSOME at room temperature helps to reduce sperm motility and it has been suggested that this also avoids sperm vacuole formation that may occur following prolonged micromanipulation at higher temperature [88].

### 8.3. Clinical Use

The initial RCTs of IMSI yielded varied results, with one demonstrating increased implantation and clinical pregnancy rates and the other finding no significant difference in clinical outcome between ICSI and IMSI in unselected patients [89,90]. Subsequent meta-analyses showed that IMSI increased clinical pregnancy rates, though the quality of some of the data was undermined by low numbers of heterogeneous patients [86,91]. On the other hand, a large multicentre RCT failed to show any benefit of IMSI regardless of sperm nuclear maturity and the extent of sperm DNA fragmentation [92]. However, a meta-analysis of IMSI in selected patients with previous implantation failure reported a significant increase in implantation and clinical pregnancy rates and reduction in miscarriage rate [93]. Nevertheless, Cochrane systematic reviews have concluded that there remains uncertainty over any benefit of IMSI on clinical pregnancy, live birth, and miscarriage rates [94,95].

### 8.4. Summary

IMSI is a technique that combines MSOME with ICSI, thereby theoretically increasing the probability of injecting DNA-intact, genetically normal spermatozoa. However, the theoretical basis of MSOME is complicated by the appearance of vacuoles during normal physiological processes such as capacitation and the acrosome reaction. Furthermore, the published evidence regarding the perceived benefit of IMSI is controversial. Therefore, well designed large multicentre RCTs of IMSI are still required before it may be considered a routine clinical practice.

## 9. Conclusions

The main sperm separation and selection techniques currently in regular use include swim-up, DGC, ZyMõt, and PICSI (with or without SpermSlow™). However, only swim-up and DGC can be considered routine techniques since they have been used to alleviate male factor infertility for the past 50 years. In contrast, ZyMõt is a relatively recent new approach, and PICSI is indicated in cases where there is an increased risk of implantation failure or miscarriage due to elevated sperm DNA fragmentation. In this respect, there is very good evidence that PICSI significantly decreases miscarriage rates. These newer advanced methods of sperm preparation for IUI, IVF, and ICSI offer great promise for the improvement of clinical outcomes in future.
